# A song of iron and oxygen: Hypoxic pulmonary vasoconstriction and gas exchange in chronic obstructive pulmonary disease

**DOI:** 10.1113/EP091078

**Published:** 2023-02-06

**Authors:** Jacob P. Hartmann, Damian M. Bailey, Ronan M. G. Berg

**Affiliations:** ^1^ Centre for Physical Activity Research Copenhagen University Hospital – Rigshospitalet Copenhagen Denmark; ^2^ Department of Clinical Physiology and Nuclear Medicine Copenhagen University Hospital – Rigshospitalet Copenhagen Denmark; ^3^ Neurovascular Research Laboratory, Faculty of Life Sciences and Education University of South Wales Pontypridd UK; ^4^ Department of Biomedical Sciences, Faculty of Health and Medical Sciences University of Copenhagen Denmark

Iron has been around since shortly after the Big Bang and has been present on planet Earth from the start, forming much of its core and crust, where it has served as a *prima essentia* of life since before the Great Oxidation Event ∼2.4 billion years ago (Bailey & Poole, 2022; Sheftel et al., 2012). Iron is involved in numerous physiological mechanisms, including hypoxic vasoconstriction, a phylogenetically ancient vascular reflex conserved across all vertebrates, thus present in fish gills and amphibian skin, in addition to the lungs of most reptiles, birds and mammals (Russell et al., 2008). In Mammalia, it manifests as hypoxic pulmonary vasoconstriction (HPV), which ensures the preservation of fetal oxygenation during placental gestation by reducing blood flow through the non‐ventilated lungs. HPV also plays important roles postnatally in relationship to several extreme physiological and clinical phenomena, helping to restore the matching of pulmonary perfusion to ventilation when hypoxic regions in the lung develop. There is a renewed interest in the mechanisms and (mal)adaptability of HPV in humans, highlighted in recent publications focused on static apnoea and high‐altitude acclimatization (Jernigan et al., 2021; Kelly et al., 2022; Subedi et al., 2022). These studies have confirmed the modulatory significance of iron on HPV, providing clues to the limits of physiological adaptation to terrestrial extremes that have translational relevance for mechanisms of disease. As outlined below, impaired HPV and changes in iron homeostasis are both prominent features in chronic obstructive pulmonary disease (COPD), and the recent studies thus shed light on putative mechanisms of impaired pulmonary gas exchange in this condition.

The site of HPV is pulmonary vascular smooth muscle (PVSMCs), notably in arterial vessels, and encompasses three interconnected steps: (1) physiological sensing of reduced alveolar (not blood) oxygen tension; (2) initial PVSMC contraction; and (3) subsequent modulation of PVSMC contraction (Smith & Schumacker, 2019). Together, these steps lead to a characteristic biphasic response, with an initial increase in pulmonary vascular resistance that peaks within 5 min and a more gradual increase that reaches a sustained maximum at 30–60 min (Figure 1a). Phase 1 consists of steps 1 and 2 and depends on the formation of free radicals and associated reactive oxygen species (ROS) that trigger PVSMC contraction through local intracellular effects and by a superoxide anion‐mediated reduction in the vascular bioavailability of NO (Figure 1b). Step 3 occurs during phase 2 and largely depends on changes in the gene expression of hypoxia‐inducible factors (HIFs), with a subsequent release of vasoactive substances within the pulmonary vasculature (Figure 1c). Of note, all three steps involve altered redox status and are thus susceptible to changes in iron homeostasis, but an apparent paradox is at play here. Thus, although both hypoxia per se and iron loading increase ‘free catalytic’ (i.e. ferrous) iron (Fe^2+^) (Bailey et al., 2022), which one would expect to enhance PVSMC contraction (Figure 1b), free catalytic iron also functions to reduce PVSMC contraction by increased degradation of HIF through effects on prolyl hydroxylase domain enzymes (Figure 1c).

**FIGURE 1:**
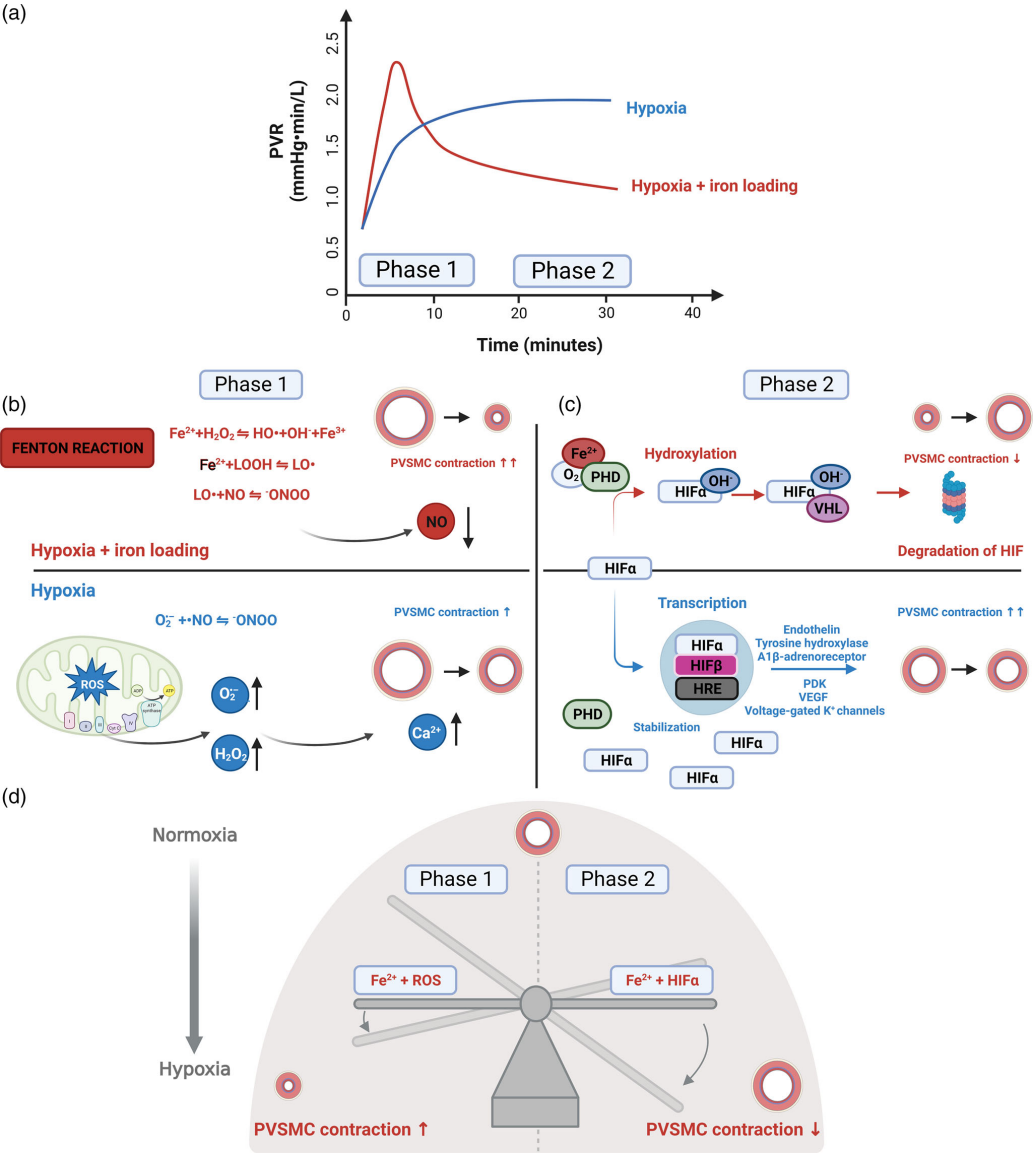
Effects of iron loading on hypoxic pulmonary vasoconstriction (HPV). (a) Time course of the change in pulmonary vascular resistance (PVR) in response to alveolar hypoxia (blue) and the presumed time course of the response upon additional iron loading (red). (b) During phase 1, hypoxia increases the formation of free radicals and associated reactive oxygen species (ROS) primarily in complex III of the mitochondria, where they then move from the intermembrane space to the cytosol, where the superoxide anion ($O_{2}^{\cdot -}$) is converted to freely diffusible hydrogen peroxide (H_2_O_2_). In pulmonary smooth muscle cells (PVSMCs), H_2_O_2_ activates phospholipase C, leading to production of inositol 1,4,5‐trisphosphate (IP_3_) and diacylglycerol (DAG), both of which facilitate Ca^2+^ release from the sarcoplasmic reticulum. The H_2_O_2_ also contributes by directly activating ryanodine receptors, and the increase in intracellular Ca^2+^ ultimately leads to contraction of the PVSMCs. Furthermore, $O_{2}^{\cdot -}$ also contributes to this by reducing the vascular bioavailability of NO through oxidative annihilation. Upon iron loading, the presence of excess ‘free catalytic’ ferrous iron (Fe^2+^) leads to increased lipid‐derived free radicals [lipid‐derived alkoxyl (LO•) and lipid hydroperoxides (LOOH)] through the Fenton reaction, further exacerbating NO scavenging and increasing PVSMC contraction in response to hypoxia. (c) Phase 2 is driven by an upregulation of hypoxia‐inducible factor (HIF) gene expression. The prolyl hydroxylase domain (PHD) enzymes regulate the stability of HIF in response to oxygen availability. During normoxia with a normal Fe^2+^ concentration, prolyl hydroxylase domain enzymes (PHDs) hydroxylate the HIFα subunit, leading to its polyubiquitination and subsequent proteasomal degradation. During hypoxia, the activity of the PHDs is reduced, leading to HIFα stabilization, initiating transcription of vasoactive moieties and upregulation of receptors augmenting vasoconstriction. Fe^2+^ serves as a cofactor for PHDs and enables them to hydroxylate HIFα despite hypoxia, thus reducing the concomitant PVSMC contraction. (d) The push and pull of free catalytic iron on HPV is illustrated by the ‘iron seesaw’. The steady‐state change in PVR in response to hypoxia upon iron loading involves an interplay between phase 1 and phase 2 mechanisms, whereby NO scavenging attributable to the Fenton reaction functions to titrate the dominant HIF‐dependent response. Figure created with BioRender.com.

In two recent studies, one from the UBC‐Nepal Expedition, conducted at The Pyramid International Laboratory at 5050 m, and the other the GLOBAL REACH 2018 study, conducted at 4300 m in Cerro de Pasco, Peru, a 220 mg i.v. iron(III)‐hydroxide sucrose infusion blunted the HPV response to ascent in lowlanders, Sherpa and Andeans (Patrician et al., 2022; Willie et al., 2021). Although ascent caused a reduction in serum iron, transferrin and transferrin saturation (Willie et al., 2021), no measurements of free catalytic iron or other measures of redox status were made in the two studies, and it remains to be documented that iron loading by this approach causes an increase in free catalytic iron in the pulmonary vascular bed. However, when considering the aforementioned ‘push and pull’ of iron homeostasis on HPV, the results might be interpreted to reflect that rather than driving the final steady‐state response, NO scavenging primarily functions hermetically to titrate the effects of HIF‐dependent pathways on pulmonary vascular resistance, to fine‐tune regional ventilation–perfusion matching (Figure 1d).

In COPD, chronic hypoxaemia is an independent predictor of death and is notoriously difficult to manage (Martinez et al., 2006). It evolves owing to airway, alveolar and pulmonary vascular abnormalities, causing an uneven distribution of alveolar ventilation relative to perfusion. In this regard, HPV functions to match perfusion to the uneven ventilation, thus preventing or alleviating hypoxaemia according to studies based on the classical multiple inert gas elimination technique conducted in patients with stable COPD of various severities (Agusti et al., 1990; Barberà et al., 1996). The lungs of stable COPD patients with resting hypoxaemia are dominated by areas with low ventilation–perfusion ratios, indicating impaired HPV (Wagner et al., 1977). This is supported by in vitro studies on pulmonary vascular specimens obtained from lungs of patients with mild‐to‐moderate and severe COPD, undergoing either lung resection or lung transplantation, in which HPV has been reported to be weaker in hypoxaemic than in non‐hypoxaemic COPD patients (Peinado et al., 2013, 2002). Furthermore, during COPD exacerbations, which are characterized by an acute and sustained worsening of symptoms, hypoxaemia is associated with greater perfusion of poorly ventilated areas (Barberà et al., 1997). These findings thus support the failure of HPV as a unifying mechanism of hypoxaemia, both in stable COPD and during acute exacerbations.

The impact of iron‐redox homeostasis on HPV remains to be investigated directly in COPD (Cloonan et al., 2017), but it is worth noting that despite both systemic anaemic and non‐anaemic iron deficiency being common in COPD, the lung tissue of COPD patients consistently exhibits increases in iron content and iron‐binding molecules (Silverberg et al., 2014). This is further exaggerated during acute exacerbations, probably driven by increased iron sequestration within the lung tissue and vasculature (Cloonan et al., 2017). Although no studies have yet reported free catalytic iron levels in blood or lung tissue, either in stable COPD or during acute exacerbations, we postulate that disrupted iron homeostasis is a likely catalyst that drives the shift from a non‐hypoxaemic to a hypoxaemic phenotype in COPD.

Collectively, the available studies at present indicate the importance of taking an integrated physiological approach, by combining findings and concepts from the extreme, the classical, the healthy and the ill, to determine precisely how iron and its ancient gaseous partner oxygen, both formed in dying stars billions of years ago, contribute to the impairment of HPV in COPD.

## AUTHOR CONTRIBUTIONS

Jacob Peter Hartmann: conception, first draft, figure and revisions. Damian M. Bailey: revisions and supervision. Ronan M. G. Berg: conception, first draft, revisions, figure and supervision. All authors approved the final version of the manuscript and agree to be accountable for all aspects of the work in ensuring that questions related to the accuracy or integrity of any part of the work are appropriately investigated and resolved. All persons designated as authors qualify for authorship, and all those who qualify for authorship are listed.

## CONFLICT OF INTEREST

None declared.

## FUNDING INFORMATION

The Centre for Physical Activity Research (CFAS) is supported by TrygFonden (grants 101390 and 20045). J.P.H. is supported by Rigshospitalet Research Foundation and HelseFonden. D.M.B. is supported by a Royal Society Wolfson Research Fellowship (#WM170007).
